# Advances and Challenges in Computational Prediction of Effectors from Plant Pathogenic Fungi

**DOI:** 10.1371/journal.ppat.1004806

**Published:** 2015-05-28

**Authors:** Jana Sperschneider, Peter N. Dodds, Donald M. Gardiner, John M. Manners, Karam B. Singh, Jennifer M. Taylor

**Affiliations:** 1 CSIRO Agriculture Flagship, Centre for Environment and Life Sciences, Perth, Western Australia, Australia; 2 CSIRO Agriculture Flagship, Black Mountain Laboratories, Canberra, Australian Capital Territory, Australia; 3 CSIRO Agriculture Flagship, Queensland Bioscience Precinct, Brisbane, Queensland, Australia; 4 University of Western Australia Institute of Agriculture, University of Western Australia, Crawley, Western Australia, Australia; McGill University, CANADA

## Fungal Effector Proteins Underpin Diverse Infection Strategies

Fungi occupy diverse environmental niches and many have evolved to live a pathogenic lifestyle, causing devastating diseases in plants and animals. The interface between host and pathogen is complex and constantly evolving. Pathogens secrete effector proteins that manipulate the host to the pathogen’s advantage. Depending on their infection strategy, fungal pathogens may deliver apoplastic effectors into the extracellular spaces and/or cytoplasmic effectors that are taken up by plant cells. Effectors have a broad functional spectrum, ranging from effectors in necrotrophic pathogens with toxic activity that cause plant cell death to avirulence (Avr) effectors in biotrophic pathogens that trigger defense responses and that the plant immune system has evolved to recognize. Molecular studies have revealed over 60 fungal effectors from different species; however, this represents only the tip of the iceberg. For example, only six effectors have thus far been characterized across three rust fungi, while more than 30 *Avr* specificities have been identified in flax rust and around 50 in each of stem rust, stripe rust, and leaf rust [1]. Similarly, over 40 *Avr* specificities occur in interactions between *Magnaporthe oryzae* and rice [2].

With the rising number of sequenced pathogen genomes, computational prediction of effector proteins holds promise as a fast and economical technique to define candidates for subsequent laboratory work. Bacterial effectors delivered to the host via dedicated pathogen-derived delivery mechanisms, such as the type III secretion system, can be predicted using machine learning approaches based on protein sequence information. In oomycetes, consensus sequence motifs implicated in host translocation, such as RXLR, can be exploited for effector prediction. However, computational effector prediction in fungi is challenging due to a lack of known protein features that are common to fungal effectors and the low number of characterized effectors for individual species, which limits the use of machine learning approaches.

## Fungal Effector Proteins Generally Lack Sequence Similarity and Conserved Sequence Motifs, but Some Might Share Structural Similarity

Fungal effector prediction is a difficult problem due to the lack of unifying sequence features or structural folds for effectors within and across species. In general, fungal effectors do not share significant sequence similarity with each other, which can be attributed to rapid divergence and host specialization. However, there are some exceptions. The *Cladosporium fulvum* Ecp6 effector contains LysM domains and has strong sequence similarity to *Magnaporthe oryzae* Slp1 and other fungal genes [3]. Furthermore, some effector proteins can have a functional annotation that suggests a role in pathogenicity, for example, the chorismate mutase effector in the biotrophic maize pathogen *Ustilago maydis* [4]. Unlike the oomycete RXLR and Crinkler families of cytoplasmic effectors, no widely conserved sequence-based motifs have thus far been identified for fungal effectors, despite suggestions of RXLR-like sequences in some fungal effectors [5]. There is sporadic evidence of conserved N-terminal sequence motifs in fungal proteins with a secretion signal. For example, effector candidates in the barley powdery mildew fungus, *Blumeria graminis* f.sp. *hordei*, share an N-terminal [YFW]xC motif within 30 amino acids of the signal peptide [6]. This motif has also been reported in some effector candidates of rust fungi, but with less positional conservation [7]. In *Fusarium*, a group of proteins share a conserved [SG]PC[KR]P motif immediately after the signal peptide [8,9]. However, these motifs have not been functionally characterized and can, thus, not be confirmed as fungal effector sequence motifs. [10]. AvrL567 and AvrM from *Melampsora lini* enter flax cells autonomously mediated by N-terminal uptake domains, however, these do not share conserved motifs or structures [11]. The C-terminal RGD sequence motif in the ToxA effector is required for wheat cell entry [10].

More subtle features other than sequence similarity may unify classes of effectors, such as conserved three-dimensional folds. For examples, many oomycete RxLR effectors share a common WY domain fold [12], while the powdery mildew [YFW]xC class effectors are predicted to share a structural fold related to ribonucleases [13]. Similar β-sandwich structures were identified in AvrL567 from *Melampsora lini* [14], the ToxA effector from *Pyrenophora tritici-repentis* [15] and in the *M*. *oryzae* effector AvrPiz-t [16], suggesting that this fold might be common in fungal effectors. Interestingly, the three-dimensional structure of the *M*. *lini* effector AvrM contains a tandem duplicated four-helical motif with similarity to the WY domain of oomycete effectors [12]. Thus, while there may be some structural conservation within certain families of fungal effectors, overall, the lack of conserved structural features suggests difficulty in exploiting these for effector prediction.

## Secreted, Small, and Cysteine-Rich: Prediction of Apoplastic Effector Repertoires from Genomes

**Fig 1:**
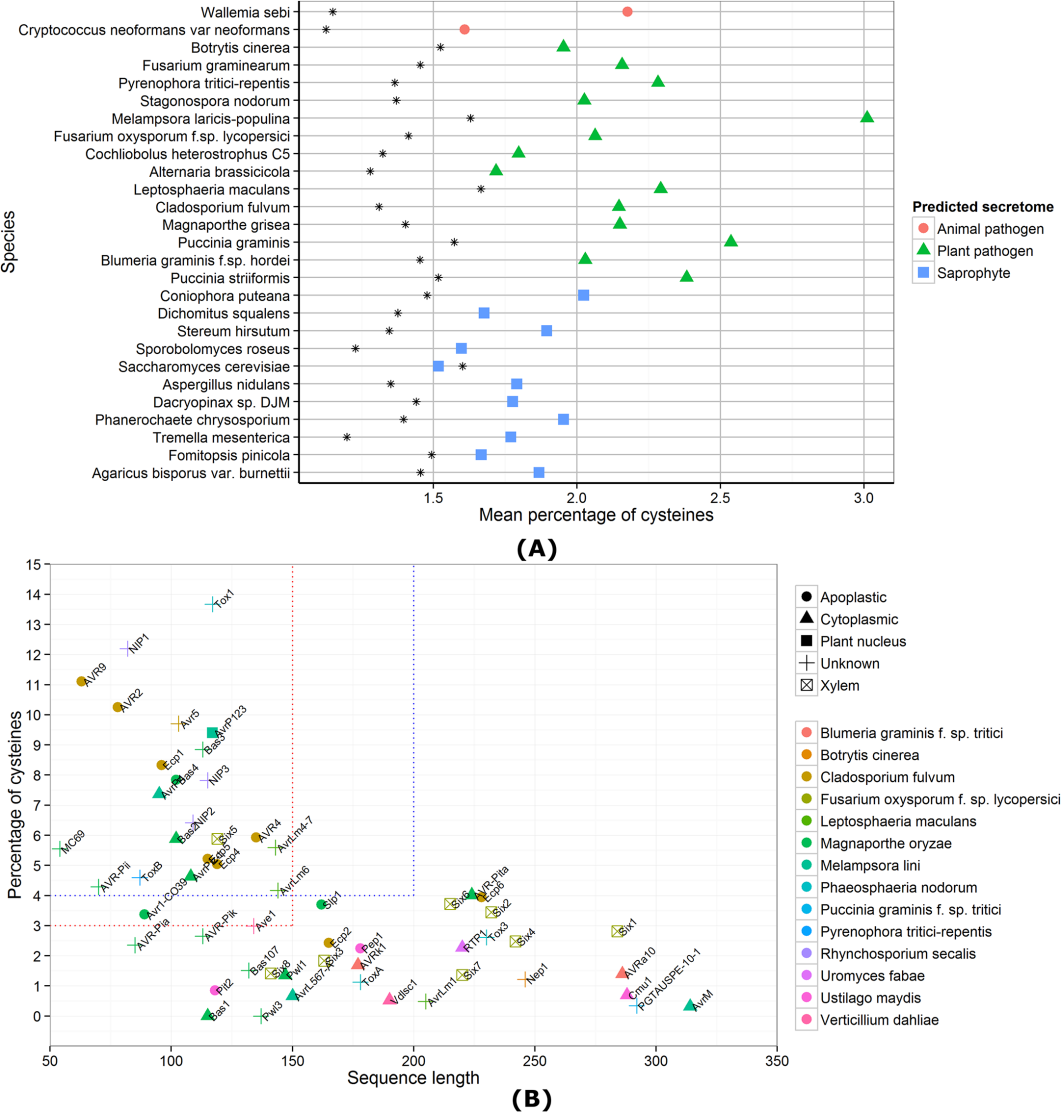
Cysteine content of predicted fungal secretomes and fungal effector properties. (A) For each species, the mean percentage of cysteines is shown for all predicted genes (as a black star) and the secretome predicted by SignalP 4.1 [31]. Apart from *S*. *cerevisiae*, all species have a higher mean percentage of cysteines in their secretomes, compared to the genome-wide mean. (B) Sequence lengths and cysteine content of known fungal effector proteins are shown. The red dotted lines indicate the criteria for small, cysteine-rich defined in Saunders et al. [21] and the blue dotted lines the criteria for small, cysteine-rich defined in Ma et al. [8]. A trend for species-specific conservation of small, cysteine-rich effectors cannot be observed. Even the *C*. *fulvum* pathogen that is known to grow extracellularly has two effectors that do not fit under the small, cysteine-rich umbrella defined by commonly used thresholds.

Given the lack of conserved sequence features, fungal effector prediction approaches have been based on relatively broad criteria, principally the presence of a secretion signal. In addition, most known fungal effectors are small in size and often rich in cysteine residues. Apoplastic effectors, in particular, often contain several disulfide bonds [17] and predicted secretomes of pathogenic fungi contain proteins with elevated levels of cysteines compared to all proteins (Fig 1A). Therefore, the criteria of small and cysteine-rich can be used to mine predicted secretomes for apoplastic effectors and reduce the number of candidates [18,19]. However, not all secreted proteins with small size and high cysteine content will have an effector function and, conversely, not all fungal effectors will be small and cysteine-rich. Many cytoplasmic effectors that are delivered into host cells are low in cysteines and of larger size, which has also been found for several apoplastic effectors (Fig 1B). For example, the AvrLm1 effector from the hemibiotrophic pathogen *Leptosphaeria maculans* that colonises the apoplast has only one cysteine [20]. Whilst the criteria of small and cysteine-rich are very valuable for screening secretomes for apoplastic effectors, they are not a one-size-fits-all solution for predicting both apoplastic and cytoplasmic effectors, and do not necessarily discriminate between these classes either. For instance, the AvrP4 and AvrP123 effectors of *M*. *lini* are small and cysteine-rich, yet are recognised by intracellular immune receptors, suggesting they are delivered to the host cytoplasm [1].

## Beyond Secreted, Small, and Cysteine-Rich: Dedicated Pipelines for Predicting Apoplastic and Cytoplasmic Effectors

Sophisticated approaches for predicting apoplastic and cytoplasmic effector candidates have emerged that do not solely rely on rules, such as a predicted secretion signal, small size, and cysteine content, but also include other lines of evidence associated with fungal effectors and are potentially powerful for predicting effector candidates without making a priori assumptions on their properties.

**Fig 2:**
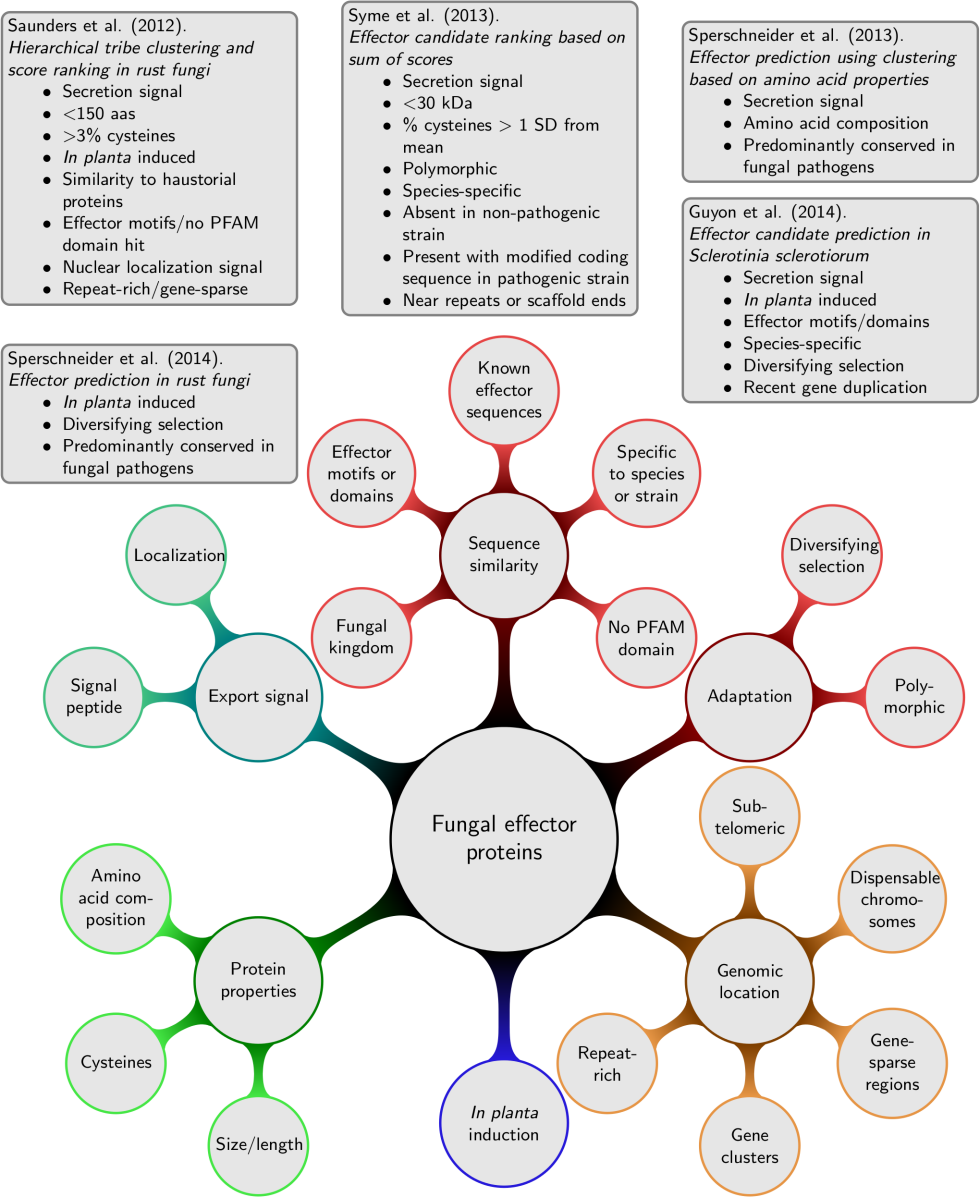
Lines of evidence that have been used for predicting fungal effector proteins and examples for fungal effector prediction pipelines.

For haustorially delivered rust fungi effectors, Saunders et al. [21] developed a ranking method according to criteria associated with experimentally verified effectors (details given in Fig 2). First, secretomes are clustered into tribes based on sequence similarity scores. Second, tribes are ranked according to the likelihood of obtaining at least the same number of proteins with the given effector property by chance. Whilst high-scoring tribes were predicted that contain likely effector families, the pipeline failed to recognize the *Puccinia graminis* f. sp. *tritici* effector PGTAUSPE-10-1 [22] as a candidate. The same pipeline was also applied to *M*. *lini* with thresholds informed by the known rust effectors, which returned 200 high priority tribes of candidate effectors [23], and to *P*. *striiformis* f. sp. *tritici* [24] combined with evidence of sequence polymorphisms and in planta expression. The combination of additional lines of evidence is very useful to reduce the set of high-priority candidates. For example, Sperschneider et al. [25] combined evidence for diversifying selection; conservation, predominantly in fungal pathogen genomes; and induction in planta and in haustoria to identify a list of 42 haustorially delivered effector candidates in *P*. *graminis* f. sp. *tritici* and successfully recovered PGTAUSPE-10-1 as the top candidate [22].

For necrotrophic pathogens, Guyon et al. [26] returned 78 effector candidates from *Sclerotinia sclerotiorum*, again, using several independent lines of evidence as shown in Fig 2. Syme et al. [27] used the sum of effector evidence scores (details in Fig 2) to rank *Stagonospora nodorum* effector candidates that are not found or that are highly divergent in a re-sequenced, non-pathogenic strain. An unsupervised exploration of fungal effector properties in cereal pathogens was performed in Sperschneider et al. [9]. Proteins that were predominantly conserved across fungal pathogens were clustered based on their amino acid properties and other sequence-derived features. This revealed putative effector clusters with enrichment in secretion signals for several fungal pathogens infecting cereals. Interestingly, some of these protein clusters are enriched in secreted proteins that have a high content of small amino acids and cysteines, whereas others are enriched in features not commonly associated with fungal effectors. This supports the view that our current knowledge of fungal effectors is still incomplete.

## Fungal Effector Prediction from Genomic Sequences: A Unified Way Forward

Whilst the full scope of fungal effectors remains a mystery, in particular for animal pathogens, characterized plant pathogen effectors have been found to be extremely versatile, targeting diverse host cell compartments and elements of the plant immune system [28]. Despite increasing insight into effector functions through molecular and structural studies, the only universal features thus far identified of fungal effectors are that they are secreted and differentially expressed during in planta infection. However, they are not necessarily computationally predicted to be secreted, as exemplified by fungal effectors that lack a predicted signal peptide and must instead use an unconventional secretion pathway [29]. Approaches for predicting fungal effectors from genomic sequences must be able to look beyond sequence-similarity-based methods and should not rely purely on selecting small and cysteine-rich proteins from the secretome as effector candidates. Classifiers that integrate other evidence for effector function, such as in planta expression data, signatures of diversifying selection, genomic features, or taxonomic information, are equally powerful and do not make a priori assumptions on effector protein properties. Future studies will be required to determine if there are structural folds or other molecular features common to fungal effectors targeting the same host cell machinery. It will be interesting to apply concepts from effector prediction in fungal plant pathogens (Fig 2) to the prediction of effectors in fungal animal pathogens to explore possible similarities [30]. Finally, an increase in the number of identified fungal effectors might enable machine learning approaches for unbiased prediction, which could lead to the discovery of protein properties common to fungal effectors.
